# A Biochemical Evaluation on Inflammatory Markers after Ureteroscopic Lithotripsy

**DOI:** 10.1155/2014/795839

**Published:** 2014-08-05

**Authors:** Ibrahim Aydin, Fevzi Nuri Aydin, Mehmet Agilli

**Affiliations:** ^1^Department of Biochemistry, Sarikamis Military Hospital, Sarikamis, Kars, Turkey; ^2^Department of Biochemistry, Sirnak Military Hospital, Sirnak, Turkey; ^3^Department of Biochemistry, Agri Military Hospital, Agri, Turkey

We have read with great interest the paper published in a recent issue of ISRN Urology by Bantis et al. [1] entitled “can tumor necrosis factor-*α* and interleukin-6 be used as prognostic markers of infection following ureteroscopic lithotripsy?” in which they report preoperative and postoperative serum interleukin-6 (IL-6) and tumor necrosis factor-*α* (TNF-*α*) levels in patients with urolithiasis. In their paper, authors report these inflammatory markers' level and speculate that high preoperative levels of serum TNF-*α* and IL-6 may indicate a predisposition for postoperative inflammation and infection. In this regard, we would like to make some comments with respect to the authors' interpretations of biochemical markers.

IL-6 is a cytokine which is secreted by macrophages and T-cells to stimulate immune response. Additionally, smooth muscle cells also produce IL-6 as a myokine, and its level is elevated in response to muscle contraction [2]. The intensity and duration of muscle contraction determine the magnitude of increase of plasma IL-6 [3]. IL-6 acts as both a proinflammatory and an anti-inflammatory cytokine. IL-6's role as an anti-inflammatory cytokine is through its inhibitory effects on TNF-*α* and IL-1 [2]. When IL-6 is signaling in monocytes or macrophages, it creates a proinflammatory response, whereas IL-6 signaling in muscle is anti-inflammatory [4].

Urolithiasis is a disease which is characterized by the formation of stones within the parts of the urinary system. As is known, these stones cause severe pain and intense muscular contractions. On the other hand, urinary stones damage the endothelium and cause the inflammatory response. In this case, there are two different mechanisms for IL-6 secretion in urolithiasis. Endothelial damage may increase the risk of infection [5], but muscular contractions cannot generate such an effect alone. For these reasons, suggesting IL-6 as an indicator of inflammatory predisposition, without determining the source of IL-6, may be objectionable.

Muscle contraction, patients' pain degree, and treatment regimen and agents which are used in general anesthesia were not stated in their paper. In order to eliminate confusion on this issue, if there is any patients complaining from pain as renal colic, these patients should be excluded, and these issues should also be stated in their paper. Furthermore, serum IL-1 levels could be useful to determining the anti-inflammatory effects of IL-6.

In conclusion, the explanation of these concerns will certainly provide clearer information for the readers.
